# Efficacy and Safety of Lucitanib in Advanced Thymic Carcinoma: A Randomized Phase II Trial

**DOI:** 10.1002/mco2.71105

**Published:** 2026-10-25

**Authors:** Wentao Fang, Caicun Zhou, Changlu Wang, Kailiang Wu, Jianhua Fu, Xiaoyan Lin, Helong Zhang, Jianhua Chen, Peng Zhang, Yiping Zhang, Peng Tang, Yulong Zheng, Yanping Liu, Xiuyi Zhi, Zhancheng Gao, Qingxia Fan, Huiming Cai, Fugen Li, Xiaoning Guo, Bo Zhang, Rongji Mu, Zhangsheng Yu

**Affiliations:** ^1^ Department of Thoracic Surgery, Shanghai East Hospital Shanghai Tongji University School of Medicine Shanghai China; ^2^ Department of Medical Oncology, Shanghai East Hospital Shanghai Tongji University School of Medicine Shanghai China; ^3^ Department of Radiation Oncology, Shanghai Chest Hospital Shanghai Jiao Tong University School of Medicine Shanghai China; ^4^ Radiotherapy Center Fudan University Shanghai Cancer Center Shanghai China; ^5^ Department of Thoracic Surgery Sun Yat‐Sen University Cancer Center Guangzhou China; ^6^ Department of Medical Oncology Fujian Medical University Union Hospital Fuzhou China; ^7^ Department of Oncology The Second Affiliated Hospital of Air Force Medical University Xi'an China; ^8^ Department of Thoracic Medicine Hunan Cancer Hospital Changsha China; ^9^ Department of Cardiothoracic Surgery Tianjin Medical University General Hospital Tianjin China; ^10^ Department of Medical Oncology Zhejiang Cancer Hospital Hangzhou China; ^11^ Department of Esophageal Oncology Tianjin Cancer Hospital Tianjin China; ^12^ Department of Medical Oncology, The First Affiliated Hospital Zhejiang University School of Medicine Hangzhou China; ^13^ Department of Oncology China Shenyang The Tenth People's Hospital Shenyang China; ^14^ Department of Thoracic Surgery Xuanwu Hospital Capital Medical University Beijing China; ^15^ Department of Respiratory Medicine Peking University People's Hospital Beijing China; ^16^ Department of Medical Oncology The First Affiliated Hospital of Zhengzhou University Zhengzhou China; ^17^ Haihe Biopharma Co., Ltd Shanghai China; ^18^ Institute of Clinical Medicine Shanghai Jiao Tong University School of Medicine Shanghai China

**Keywords:** advanced recurrent or metastatic thymic carcinoma, AL3810, lucitanib, tyrosine kinase inhibitor

## Abstract

Advanced thymic carcinoma is a rare, aggressive malignancy with limited treatment options after chemotherapy failure. This randomized, double‐blind, placebo‐controlled trial (AL3810‐202) aimed to evaluate lucitanib, a novel, orally potent tyrosine kinase inhibitor targeting VEGFR1‒3, FGFR1‒3, and PDGFRα/β in patients with advanced thymic carcinoma progressing after chemotherapy. Sixty‐eight patients were randomized (2:1) to receive 10 mg lucitanib daily (*n* = 46) or placebo (*n* = 22). Although this study did not meet its primary endpoint of independent review committee (IRC)‐assessed progression‐free survival (PFS) (median 5.8 vs. 3.7 months; hazard ratio [HR]: 0.60; *p* = 0.1050), the prespecified log‐rank test may have underestimated the treatment effect due to an observed non‐proportional hazards pattern. Subsequent restricted mean survival time analysis demonstrated a statistically significant PFS benefit (*p* < 0.05), which was further supported by significantly prolonged investigator‐assessed PFS (6.6 vs. 1.9 months; HR: 0.53; *p* = 0.0306). The most common grade 3 treatment‐related adverse events (TRAEs) were hypertension (50%), proteinuria (15.2%), and thrombocytopenia (8.7%), with no grade 4/5 events. Considering the totality of the evidence and an acceptable safety profile, lucitanib may confer a meaningful clinical benefit for patients with advanced thymic carcinoma, warranting further investigation.

## Graphical abstract

In this randomized phase II trial, 68 patients with advanced thymic carcinoma after chemotherapy received lucitanib (*n* = 46) or placebo (*n* = 22). Median PFS was 5.8 versus 3.7 months by IRC (HR: 0.60; *p* = 0.105) and 6.6 versus 1.9 months by investigators (HR: 0.53; *p* = 0.031). Hypertension was the most common grade 3 TRAE (50%). Lucitanib showed clinically meaningful activity with manageable safety.

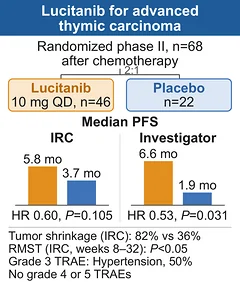

**Abbreviations**
HR: hazard ratio
IRC: independent review committee
PFS: progression‐free survival
TRAE: treatment‐related adverse event

## 1 Introduction

Thymic carcinoma is an extremely rare malignant tumor, with an estimated incidence of approximately 0.02‒0.06 per 100,000 persons [1, 2, 3], and nearly 30% of patients present with advanced disease at the time of diagnosis [1]. The disease is highly aggressive and associated with a poor prognosis, with 5‐year survival rates of approximately 24%–42% for stage IVa disease and 17%–30% for stage IVb disease [4]. Platinum‐based chemotherapy is recommended as first‐line treatment for advanced thymic carcinoma. However, following disease progression, no standard of care has been established yet, and available treatment options remain limited [4, 5].

Alternative systemic therapies include additional cytotoxic chemotherapy, immune checkpoint inhibitors (ICIs), and molecularly targeted therapies; however, evidence supporting these approaches is mainly derived from small sample, single‐arm phase II studies, with limited prospective comparative data. Second‐line chemotherapy regimens, including platinum‐based rechallenge, pemetrexed, gemcitabine‐based combinations, and other cytotoxic agents, generally provide modest efficacy, with reported response rates ranging from approximately 15% to 40% and median progression‐free survival (PFS) of around 6 months [4, 6]. ICIs have demonstrated clinical activity in thymic carcinoma, but their use requires caution because of the risk of severe immune‐related adverse events (AEs), and reliable predictive biomarkers remain lacking [6]. Given these therapeutic limitations, targeting angiogenic pathways has gained increasing attention, as angiogenesis plays a critical role in thymic carcinogenesis.

Vascular endothelial growth factor A (VEGF‐A) and its receptors, VEGFR1 and VEGFR2, which are frequently overexpressed in thymic carcinoma, play key roles in both physiologic and pathologic angiogenesis. In addition, activation of VEGFR2 and platelet‐derived growth factor receptor (PDGFR)‐α has been documented in thymic carcinoma [1, 7]. Tyrosine kinase inhibitors (TKIs) such as sunitinib, which targets VEGFR, KIT, and PDGFR [8], and lenvatinib, which inhibits VEGFR, fibroblast growth factor receptor (FGFR), c‐KIT, and other kinases [1], have demonstrated clinical activity in this setting. In phase 2 single‐arm studies, sunitinib and lenvatinib achieved objective response rates (ORRs) of 26% and 38%, respectively, in patients with advanced thymic carcinoma who had disease progression after first‐line platinum‐based chemotherapy [1, 8]. On the basis of the REMORA study, lenvatinib has been approved for the treatment of thymic carcinoma in Japan; however, all patients (100%) required dose reductions due to AEs, with grade 3 or higher treatment‐related AEs reported in a substantial proportion (e.g., grade 3 hypertension in 64%), which required frequent dose modifications and led to treatment discontinuation in 17% of patients [1].

Lucitanib (AL3810) is a novel, orally administered TKI that selectively targets VEGFR1‒3, FGFR1‒3, and PDGFRα/β. It exhibited encouraging antitumor efficacy for advanced thymic carcinoma in the first‐in‐human study in patients with advanced solid tumors and at the same time, showed a more favorable safety profile compared with the existing drugs in this class. Here, we report the results of a randomized, double‐blind, placebo‐controlled phase 2 trial (AL3810‐202) evaluating the efficacy and safety of lucitanib in patients with advanced recurrent or metastatic thymic carcinoma.

## 2 Results

### 2.1 Baseline Characteristics

Between January 10, 2019, and September 6, 2021, a total of 68 patients were enrolled, with 46 assigned to the lucitanib group and 22 to the placebo group. Most baseline characteristics were generally balanced between the two groups (Table 1). The median age of the patients was 55 years (range: 28‒74 years); 61.8% were men, and 88.2% had squamous cell carcinoma. All patients had stage IV disease according to the Masaoka‒Koga classification and had documented metastatic disease at screening. With respect to prior treatment, more than half of the patients had undergone surgery, and all had received radiotherapy. In addition, 95.5% (*n* = 64) of patients had received at least one platinum‐based chemotherapy regimen, and 41.2% (*n* = 28) had received two or more lines of chemotherapy. Owing to the small sample size, some baseline imbalances were observed; specifically, the median time from initial diagnosis to randomization and the median time from initial metastasis to randomization were shorter in the lucitanib group than in the placebo group (Table 1).

**TABLE 1:** Baseline demographic and disease characteristics (full analysis set).

Variables	Lucitanib group (*n* = 46)	Placebo group (*n* = 22)	Total (*N* = 68)
Age (years)	56.0 (28, 74)	50.5 (39, 69)	55.0 (28, 74)
Sex, *n* (%)			
Male	30 (65.2)	12 (54.5)	42 (61.8)
Female	16 (34.8)	10 (45.5)	26 (38.2)
Weight (kg)	64.35 (41.6, 102.0)	60.00 (50.0, 80.0)	60.50 (41.6, 102.0)
ECOG performance status, *n* (%)			
0	2 (4.3)	1 (4.5)	3 (4.4)
1	43 (93.5)	20 (90.9)	63 (92.6)
2	1 (2.2)	1 (4.5)	2 (2.9)
Histological type, *n* (%)			
Squamous cell carcinoma	42 (91.3)	18 (81.8)	60 (88.2)
Mucoepidermoid carcinoma	0	1 (4.5)	1 (1.5)
Sarcomatoid carcinoma	1 (2.2)	0	1 (1.5)
Adenocarcinoma	1 (2.2)	0	1 (1.5)
Undifferentiated carcinoma	1 (2.2)	2 (9.1)	3 (4.4)
Others	1 (2.2)	1 (4.5)	2 (2.9)
Stage in screening period (Masaoka‒Koga classification), *n* (%)			
IVa	1 (2.2)	1 (4.5)	2 (2.9)
IVb	45 (97.8)	21 (95.5)	66 (97.1)
Time from initial diagnosis to randomization (months)	17.35	29.95	19.25
Presence of metastases, *n* (%)	46 (100.0)	22 (100.0)	68 (100.0)
Lung	29 (63.0)	14 (63.6)	43 (63.2)
Pleura	23 (50.0)	15 (68.2)	38 (55.9)
Lymph nodes	25 (54.3)	10 (45.5)	35 (51.5)
Bone	18 (39.1)	11 (50.0)	29 (42.6)
Liver	17 (37.0)	7 (31.8)	24 (35.3)
Pleural effusion	3 (6.5)	2 (9.1)	5 (7.40
Kidney	0	2 (9.1)	2 (2.9)
Brain	1 (2.2)	0	1 (1.5)
Breast	1 (2.2)	0	1 (1.5)
Soft tissue	1 (2.2)	0	1 (1.5)
Peritoneum	0	1 (4.5)	1 (1.5)
Skin	0	1 (4.5)	1 (1.5)
Others	12 (26.1)	10 (45.5)	22 (32.4)
Time from initial metastases to randomization (months)	7.25	27.50	12.90
Previous surgery, *n* (%)	29 (63.0)	10 (45.5)	39 (57.4)
Previous radiotherapy, *n* (%)	46 (100.0)	22 (100.0)	68 (100.0)
Previous bevacizumab, *n* (%)	2 (4.3)	3 (13.6)	5 (7.4)
Previous systemic chemotherapy treatment, *n* (%)			
First line	31 (67.4)	8 (36.4)	39 (57.4)
Second line	12 (26.1)	11 (50.0)	23 (33.8)
Third line and subsequent lines	2 (4.3)	3 (13.6)	5 (7.4)
Chemotherapy received in the first‐line setting, *n* (%)			
Platinum*	43 (95.6)	21 (95.5)	64 (95.5)
Platinum plus taxanes†	33 (76.7)	16 (76.2)	49 (76.6)

Abbreviation: ECOG, Eastern Cooperative Oncology Group.

*Percentages are based on evaluable patients who received first‐line chemotherapy (*n* = 45 in the lucitanib group, *n* = 22 in the placebo group, and *n* = 67 in total). †Percentages are based on patients who received platinum‐based first‐line chemotherapy (*n* = 43 in the lucitanib group, *n* = 21 in the placebo group, and *n* = 64 in total).

At the data cutoff date of September 6, 2021, the median follow‐up time was 20.67 months (range: 19.02‒25.46) in the lucitanib group, 21.42 months (range: 16.30‒26.61) in the placebo group, and 21.42 months (range: 19.19‒25.49) in the overall population. At that time, five patients (11%) in the lucitanib group were still receiving treatment, whereas no patients in the placebo group remained on study treatment. The median duration of treatment was 24.2 weeks in the lucitanib group and 8.2 weeks in the placebo group. Disease progression was the most common reason for treatment discontinuation, occurring in 27 patients (58.7%) in the lucitanib group and 17 patients (77.3%) in the placebo group.

### 2.2 Survival Outcomes

The primary analysis was based on 52 PFS events assessed by the independent review committee (IRC), including 38 events in the lucitanib group and 14 in the placebo group, and on 54 PFS events assessed by investigators, including 37 events in the lucitanib group and 17 in the placebo group, among the 68 enrolled patients. In the prespecified analysis set, the IRC‐assessed median PFS was 5.8 months (95% confidence interval [CI]: 4.07‒7.29) in the lucitanib group and 3.7 months (95% CI: 0.99‒7.29) in the placebo group (hazard ratio [HR]: 0.60; 95% CI: 0.32‒1.13; log‐rank *p* = 0.1050) (Figure 1A). The investigator‐assessed median PFS was 6.6 months (95% CI: 5.45‒7.49) in the lucitanib group and 1.9 months (95% CI: 0.92‒9.23) in the placebo group (HR: 0.53; 95% CI: 0.29‒0.96; log‐rank *p* = 0.0306) (Figure 1B).

**FIGURE 1:**
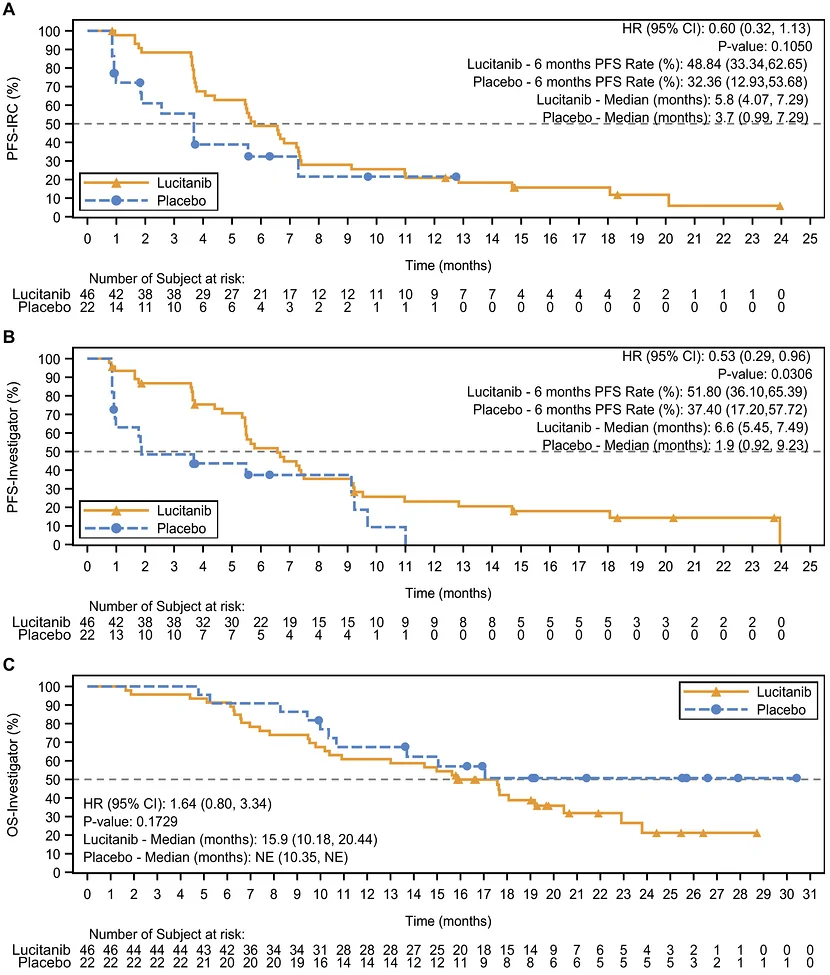
Kaplan‒Meier analysis of progression‐free survival and overall survival. (A) Progression‐free survival assessed by the independent review committee (IRC). The median progression‐free survival was 5.8 months in the lucitanib group and 3.7 months in the placebo group. (B) Progression‐free survival assessed by investigators. The median progression‐free survival was 6.6 months in the lucitanib group and 1.9 months in the placebo group. (C) Overall survival. The median overall survival was 15.9 months in the lucitanib group and was not reached (NE) in the placebo group. Survival curves were compared between treatment groups using the log‐rank test, and hazard ratios (HRs) with corresponding 95% confidence intervals (CIs) were estimated with the Cox proportional hazards model.

At the data cutoff date of September 6, 2021, 41 patients (60.3%) had died, including 31 patients (67.4%) in the lucitanib group and 10 patients (45.5%) in the placebo group. Median overall survival (OS) was 15.9 months (95% CI: 10.18‒20.44) in the lucitanib group and was not reached (95% CI: 10.35‒not estimable [NE]) in the placebo group (Figure 1C). The HR for death between treatment groups was 1.64 (95% CI: 0.80‒3.34; *p* = 0.1729). Subsequent anticancer therapies were administered to 27 patients (58.7%) in the lucitanib group and 18 patients (81.8%) in the placebo group (Table S1). Among patients who died, disease progression was the most common reported cause of death in both groups (Table S2). Subgroup analyses, as summarized in forest plots, consistently demonstrated a PFS benefit favoring lucitanib over placebo, with HRs of less than 1 across all evaluable subgroups (Figure 2A,B).

**FIGURE 2:**
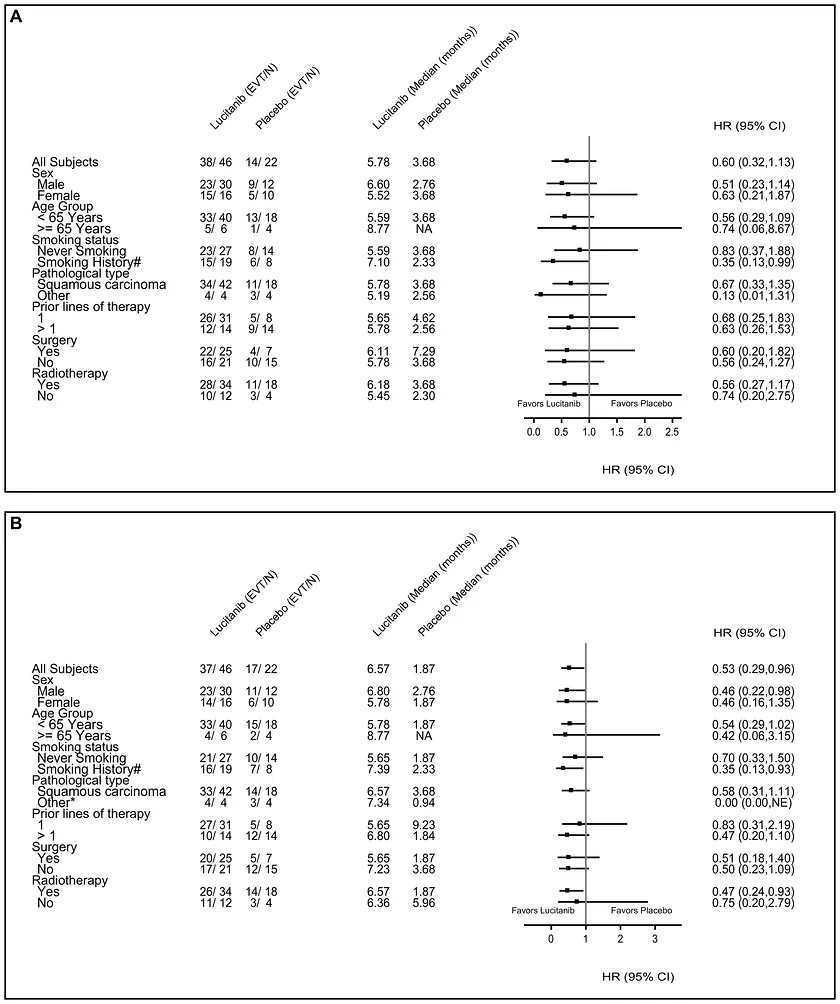
Subgroup analyses of progression‐free survival assessed by the independent review committee (IRC) and investigators. Forest plots summarize subgroup analyses of progression‐free survival as assessed by the IRC (A) and investigators (B), comparing the lucitanib group with the placebo group. Across subgroups, the analyses consistently demonstrated a progression‐free survival benefit favoring lucitanib, with hazard ratios (HRs) of less than 1. The symbol ‘#’ indicates patients with a history of smoking, including current and former smokers. An asterisk ‘*’ indicates subgroups in which the HR and corresponding confidence interval could not be calculated because of an insufficient number of progression‐free survival events. EVT denotes event and *N* denotes number of patients.

In the prespecified multivariable Cox proportional hazards regression analyses, treatment assignment and selected baseline clinical factors were evaluated for their associations with PFS. For IRC‐assessed PFS, lucitanib was associated with a numerical reduction in the risk of progression or death compared with placebo (HR: 0.58; 95% CI: 0.30‒1.11; *p* = 0.1008). For investigator‐assessed PFS, lucitanib significantly reduced the risk of progression or death (HR: 0.49; 95% CI: 0.26‒0.90; *p* = 0.0220) (Table S3). To further explore potential factors influencing PFS and assist interpretation of the primary endpoint findings, exploratory post hoc multivariable Cox regression analyses were performed. After adjustment for time from initial diagnosis to randomization, the treatment effect remained consistent for both IRC‐assessed PFS (HR: 0.45; 95% CI: 0.24‒0.86; *p* = 0.0161) and investigator‐assessed PFS (HR: 0.44; 95% CI: 0.24‒0.80; *p* = 0.0078) (Table S3).

Although a very low HR was observed for IRC‐assessed PFS, it was not statistically significant due to both the small sample size and the violation of the PH assumption (*p* = 0.0230). To further evaluate the PFS benefit of lucitanib compared with placebo, we performed a restricted mean survival time (RMST) analysis as a key sensitivity approach. In this study, RMST analysis was conducted at milestones of weeks 8, 16, 24, 32, 40, and 48. As shown in Figure 3, the RMST estimates demonstrated a significantly prolonged PFS benefit for lucitanib versus placebo at weeks 8, 16, 24, and 32 (all *p* < 0.05). To further assess the robustness of the primary IRC‐assessed PFS results in the context of a violated proportional hazards assumption, additional post hoc sensitivity analyses were performed using alternative statistical approaches. First, several weighted log‐rank tests were applied to place greater emphasis on early and intermediate PFS events, which are particularly relevant in aggressive malignancies with limited survival. These analyses consistently demonstrated statistically significant PFS benefit favoring lucitanib over placebo across all four weighting schemes, including the modified Peto‒Peto test (*p* = 0.0193), Peto‒Peto test (*p* = 0.0202), Tarone‒Ware test (*p* = 0.0342), and Gehan‒Breslow test (*p* = 0.0127). In addition, Bayesian survival analyses were conducted to quantify the probability of treatment benefit without reliance on frequentist hypothesis testing. Bayesian log‐normal regression yielded a posterior probability of approximately 100% that lucitanib was superior to placebo in prolonging PFS (Pr[*β*_1_ > 0 | *D*] = 1) (Figure S1A), while Bayesian Weibull regression demonstrated a 96.10% posterior probability of an HR below 1 (Pr[HR < 1 | *D*] = 0.961) (Figure S1B). Collectively, these complementary analyses consistently supported a clinically meaningful PFS advantage with lucitanib and reinforced the robustness of the primary efficacy findings despite the small sample size inherent to rare‐tumor trials.

**FIGURE 3:**
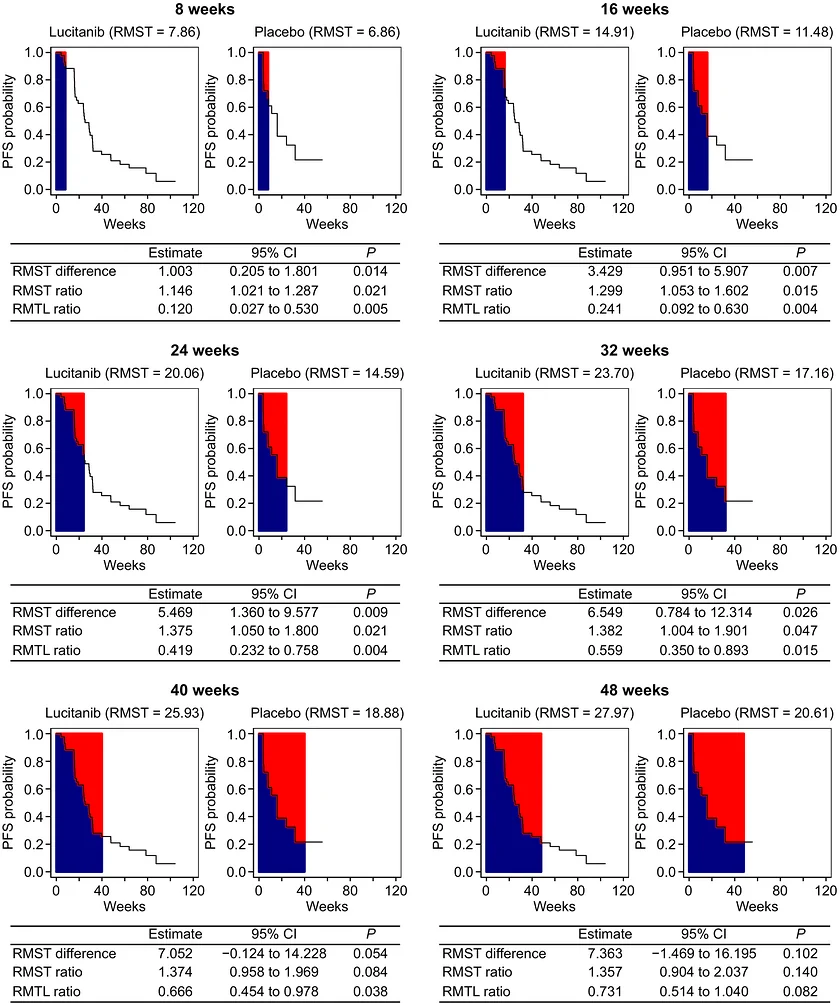
Restricted mean survival time (RMST) analysis of progression‐free survival assessed by the independent review committee. RMST was used to estimate average progression‐free survival at prespecified time points. The RMST analyses showed a significantly longer progression‐free survival with lucitanib than with placebo at weeks 8, 16, 24, and 32 (*p* < 0.05). The blue shaded area represents the RMST, with larger areas indicating longer progression‐free survival in the lucitanib group. The red shaded area represents the restricted mean time lost (RMTL), with smaller areas indicating longer progression‐free survival. CI denotes confidence interval.

### 2.3 Tumor Response Outcomes

Additionally, the lucitanib group also exhibited better ORR, disease control rate (DCR) and shrinkage rate of target lesion than the placebo group, as assessed by both the IRC and investigator (Figure 4 and Table S4). According to IRC assessment, a substantially higher proportion of patients in the lucitanib group experienced tumor shrinkage compared with the placebo group, with target lesion reduction observed in 82% (36/44) of patients receiving lucitanib versus 36% (8/22) of those receiving placebo (Figure 4A). Consistent findings were observed in the investigator assessment, in which 80% (36/45) of patients treated with lucitanib demonstrated tumor shrinkage, compared with 27% (6/22) in the placebo group (Figure 4B). Based on IRC assessment, the ORR and DCR in the lucitanib group were 15.2% (95% CI: 6.3‒28.9) and 82.6% (95% CI: 68.6‒92.2), respectively, with a median duration of response (DoR) of 7.1 months (95% CI: 3.48‒10.71 months). According to investigator assessment, the ORR and DCR in the lucitanib group were 13.0% (95% CI: 4.9‒26.3) and 84.8% (95% CI: 71.1‒93.7), respectively, with a median DoR in the lucitanib group was 6.5 months (95% CI: 3.68‒NE). No objective responses were observed in the placebo group. Patients treated with lucitanib demonstrated sustained tumor control over time, with most patients experiencing tumor shrinkage or stabilization during treatment, whereas tumor control was less frequently observed in the placebo group (Figure 4C,D).

**FIGURE 4:**
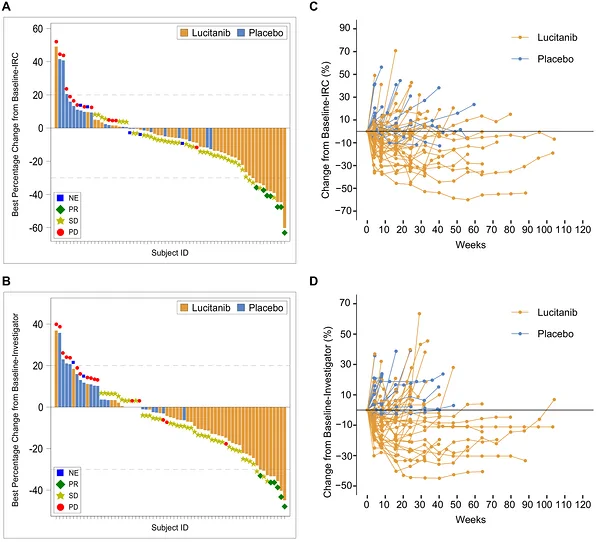
Tumor response assessed by the independent review committee (IRC) and investigators. Waterfall plots show the maximum percentage change from baseline in the sum of target lesion diameters, as assessed by the IRC (A) and investigators (B). According to IRC assessment, tumor shrinkage was observed in 82% of patients in the lucitanib group and 36% of patients in the placebo group. According to investigator assessment, tumor shrinkage was observed in 80% of patients in the lucitanib group and 27% of patients in the placebo group. Spider plots show the percentage change from baseline over time in the sum of target lesion diameters according to IRC (C) and investigator (D) assessments. Baseline tumor assessments were performed before initiation of study treatment, and subsequent tumor assessments were performed according to the study protocol until disease progression, treatment discontinuation, or the end of follow‐up. Each line represents an individual patient.

### 2.4 Safety

The median duration of lucitanib exposure was 24.2 weeks (range: 1.9‒110.7), with a median cumulative dose of 1593.75 mg (range: 130.0‒7300.0). The mean relative dose intensity was 87.5%, and 32 patients (69.6%) maintained a relative dose intensity of 80% or higher. Dose modifications occurred in 29 patients (63.0%), including dose reductions in 21 patients (45.7%), primarily due to AEs (43.5%). The median time to first dose reduction was 102 days, and the median cumulative duration of dose interruption was 18 days (Table S5).

The safety population included 46 patients who received at least one dose of lucitanib at 10 mg once daily and 22 patients who received at least one dose of placebo. The most common treatment‐related adverse events (TRAEs) of any grade in the lucitanib group, as compared with the placebo group, were proteinuria (73.9% vs. 22.7%), hypertension (71.7% vs. 9.1%), hypothyroidism (58.7% vs. 4.5%), and thrombocytopenia (32.6% vs. 4.5%). The most frequently reported grade 3 TRAEs in the lucitanib group were hypertension (50.0%), proteinuria (15.2%), and thrombocytopenia (8.7%). No grade 3 TRAEs were reported in the placebo group, and no grade 4 or 5 TRAEs occurred in either group (Table 2).

**TABLE 2:** Treatment‐related adverse events by preferred term (safety analysis set).

Events, *n* (%)	Lucitanib group (*n* = 46)		Placebo group (*n* = 22)	
	Any grade	Grade 3	Any grade	Grade 3
Proteinuria	34 (73.9)	7 (15.2)	5 (22.7)	0
Hypertension	33 (71.7)	23 (50.0)	2 (9.1)	0
Hypothyroidism	27 (58.7)	1 (2.2)	1 (4.5)	0
Thrombocytopenia	15 (32.6)	4 (8.7)	1 (4.5)	0
Blood thyroid‐stimulating hormone increased	13 (28.3)	0	0	0
Aspartate aminotransferase increased	12 (26.1)	1 (2.2)	3 (13.6)	0
Weight decreased	10 (21.7)	0	2 (9.1)	0
Hyperbilirubinemia	10 (21.7)	1 (2.2)	1 (4.5)	0
Alanine aminotransferase increased	9 (19.6)	0	2 (9.1)	0
Hyperuricemia	9 (19.6)	2 (4.3)	2 (9.1)	0
Leukopenia	9 (19.6)	0	1 (4.5)	0
Hypertriglyceridemia	9 (19.6)	2 (4.3)	1 (4.5)	0
Decreased appetite	9 (19.6)	1 (2.2)	1 (4.5)	0
Diarrhea	9 (19.6)	1 (2.2)	0	0
Hypercholesterolemia	8 (17.4)	0	0	0
Hyperglycemia	8 (17.4)	0	0	0
Anemia	7 (15.2)	1 (2.2)	3 (13.6)	0
Asthenia	7 (15.2)	0	2 (9.1)	0
Hematuria	6 (13.0)	0	1 (4.5)	0
Hyponatremia	5 (10.9)	2 (4.3)	0	0
Abdominal pain	5 (10.9)	0	0	0
Arthralgia	5 (10.9)	1 (2.2)	0	0
Blood urea increased	5 (10.9)	0	0	0
Neutropenia	5 (10.9)	0	0	0

*Note*: The listed treatment‐related adverse events were reported in at least 10% of the patients for any grade in the lucitanib group.

No grade 4/5 treatment‐related adverse events were observed in each group.

Treatment‐related serious AEs occurred in 10 patients (21.7%) in the lucitanib group, whereas no treatment‐related serious AEs were observed in the placebo group. No deaths related to TRAEs occurred in either group.

AEs leading to dose interruption occurred in 19 patients (41.3%) receiving lucitanib and in three patients (13.6%) receiving placebo. Dose reductions due to AEs were required in 20 patients (43.5%) in the lucitanib group, as compared with one patient (4.5%) in the placebo group. Treatment discontinuation due to AEs occurred in six patients (13.0%) in the lucitanib group and in one patient (4.5%) in the placebo group.

In an exploratory analysis according to dose modification status, dose modification was not associated with impaired clinical efficacy. Patients who underwent dose modification maintained favorable efficacy outcomes, with a median IRC‐assessment PFS of 7.23 versus 2.87 months (HR: 0.35; 95% CI: 0.18‒0.67), investigator‐assessment PFS of 7.49 versus 4.67 months (HR: 0.35; 95% CI: 0.17‒0.70), and a numerically longer median OS of 17.68 versus 9.53 months (HR: 0.57; 95% CI: 0.27‒1.17) compared with those without dose modifications (Figure S2).

## 3 Discussion

In this randomized, double‐blind, placebo‐controlled phase 2 trial, lucitanib demonstrated promising antitumor activity with an acceptable safety profile in patients with advanced recurrent or metastatic thymic carcinoma who had failed at least one line of chemotherapy. Treatment with lucitanib was associated with a consistent improvement in PFS compared with placebo, as assessed by both investigators and an IRC, supported by concordant findings across multiple sensitivity analyses. The safety profile was consistent with on‐target antiangiogenic effects and was generally manageable with dose modification, without evidence of unexpected toxic effects. Collectively, these findings provide the first randomized evidence supporting the efficacy of lucitanib in patients with limited treatment alternatives after chemotherapy failure.

In this study, treatment with lucitanib resulted in promising efficacy in a population of patients with advanced recurrent or metastatic thymic carcinoma who had limited therapeutic options after chemotherapy failure. Median PFS approached 6 months with lucitanib, corresponding to an approximately 40%‒47% reduction in the risk of disease progression or death compared with placebo, as assessed by both investigators and an IRC. This benefit was consistent across prespecified subgroups and was supported by concordant improvements in secondary efficacy measures, including 6‐month PFS rates, ORR, DCR, and depth of tumor shrinkage, all of which favored lucitanib. The prespecified confirmatory hypothesis was not met, as the primary endpoint of IRC‐assessed PFS did not achieve statistical significance according to the planned log‐rank analysis. This primary analysis was designed under the assumption of proportional hazards; however, the observed survival curves exhibited a non‐proportional hazards pattern. Therefore, the efficacy findings from additional sensitivity analyses should be interpreted as supportive rather than confirmatory.

Although direct comparisons across trials should be interpreted cautiously, the efficacy observed with lucitanib in the present randomized study is notable when contextualized against prior single‐arm investigations of multitargeted TKIs in a similar clinical setting. In the REMORA trial, lenvatinib achieved an ORR of 38.1% and a median PFS of 9.3 months, whereas sunitinib demonstrated an ORR of 26.1% and a median PFS of 7.2 months in chemotherapy‐refractory thymic carcinoma [1]. By contrast, lucitanib was associated with a median PFS of approximately 6 months and an ORR of approximately 15% in the current study. However, patients enrolled in this trial had substantially more advanced disease and poorer baseline clinical characteristics than those in the lenvatinib and sunitinib studies [1, 8], including a markedly higher proportion with Eastern Cooperative Oncology Group (ECOG) performance status scores of 1 or greater (95.7% vs. 69% and 76%, respectively), universal stage IV disease (100% vs. 83% in REMORA), and significantly greater exposure to prior local therapies, including surgery (63% vs. 33%) and radiotherapy (100% vs. 40%). These adverse prognostic features are well recognized to attenuate treatment response and survival outcomes. Importantly, unlike prior studies, the present trial incorporated a randomized, placebo‐controlled design, allowing for a direct estimation of treatment effect; within this rigorous framework, lucitanib reduced the risk of disease progression or death by approximately 40%‒47% relative to placebo. Taken together, these findings suggest that the antitumor activity of lucitanib is clinically meaningful despite a more refractory study population.

Although exploratory, the subgroup analyses suggest biologically plausible heterogeneity in treatment benefit with lucitanib. Patients with a history of smoking derived greater benefit, with consistent reductions in the risk of progression observed in both IRC and investigator assessments (median PFS: 7.10 vs. 2.33 months and 7.39 vs. 2.33 months, respectively; HR: 0.35 in both analyses). Tobacco exposure has been associated with enhanced angiogenic signaling, including upregulation of VEGF‐related pathways and endothelial activation, as well as distinct molecular and microenvironmental features in squamous carcinomas, which may increase dependence on angiogenesis‐driven tumor growth and thereby augment sensitivity to VEGFR and FGFR inhibition [9]. Similarly, improved efficacy was observed among patients who had received prior radiotherapy, particularly in the investigator‐assessed analysis (median PFS: 6.57 vs. 1.87 months; HR: 0.47). Radiotherapy is known to induce hypoxia and vascular injury, leading to compensatory activation of proangiogenic pathways that may promote tumor regrowth; inhibition of these pathways could therefore be particularly effective in this context [10, 11]. Together, these findings are hypothesis‐generating and warrant further investigation incorporating molecular and microenvironmental correlates to better define patient subsets most likely to benefit from lucitanib.

Although lucitanib demonstrated a clinically meaningful improvement in PFS, OS results should be interpreted cautiously. In this study, no definitive OS benefit was demonstrated, and median OS was not reached in the placebo group. Several factors may have influenced the OS findings, including the limited number of death events, subsequent treatment patterns, and the clinical course after disease progression. Crossover between treatment groups was not permitted; however, subsequent anticancer therapies were administered more frequently in the placebo group, including chemotherapy, immunotherapy, and targeted therapies, which may have contributed to subsequent disease control and influenced OS comparisons. Therefore, while PFS findings support the antitumor activity of lucitanib, the OS results require cautious interpretation and further validation.

Lucitanib demonstrated a favorable and clinically manageable safety profile. Unlike ICIs such as pembrolizumab, which are associated with potentially severe immune‐related AEs, lucitanib did not induce characteristic immune‐mediated toxic effects in this study. The overall safety profile was dominated by expected on‐target antiangiogenic toxicities, including hypertension and proteinuria, which were generally manageable with standard supportive measures and dose modification. Importantly, the incidence of hand‒foot syndrome and stomatitis was notably low (≤5% and none reported, respectively), contrasting sharply with rates reported for lenvatinib in the REMORA trial (hand‒foot syndrome, 69.0%) and for sunitinib (stomatitis, 65.0%, with 20.0% grade ≥3) [1, 8]. These differences were accompanied by fewer dose reductions with lucitanib than with either comparator, suggesting improved tolerability. Mechanistically, the development of hand‒foot skin reactions is thought to require both direct keratinocyte toxicity and VEGFR2‐mediated vascular injury. In this context, prior studies have shown that lenvatinib exhibits rapid binding and slow dissociation from VEGFR2 [12], while sunitinib and sorafenib can directly induce keratinocyte apoptosis or intracellular drug accumulation, thereby exacerbating epithelial toxicity [13, 14]. By contrast, lucitanib may exert less direct cytotoxicity toward keratinocytes or mucosal epithelium or demonstrate reduced intracellular accumulation, potentially accounting for the lower incidence of cutaneous and mucosal AEs observed. Although these hypotheses require experimental validation, the consistency of this safety pattern across other solid tumor studies further supports a distinct and favorable tolerability profile for lucitanib.

Treatment management strategies are important for antiangiogenic TKIs. In the phase II REMORA trial, all 42 patients receiving lenvatinib required dose reductions due to AEs, and 84% experienced grade ≥3 AEs [15]. Similarly, real‐world studies reported high rates of dose modification with lenvatinib, with 84% and 100% of patients requiring treatment adjustments, respectively [16, 17]. In the present study, although dose modifications occurred in 63.0% of patients receiving lucitanib, most patients maintained prolonged treatment exposure, with a median treatment duration of 24.2 weeks and a mean relative dose intensity of 87.5%. Crucially, exploratory analyses indicated that dose modifications did not adversely affect PFS or OS. Instead, timely dose adjustments appeared to facilitate long‐term treatment persistence, thereby enabling patients to maintain therapeutic benefit over time.

Increasing evidence indicates that antiangiogenic agents may enhance the efficacy of ICIs by remodeling the tumor microenvironment, including vascular normalization, improved immune cell infiltration, and restoration of antitumor immune activity [6]. This biological rationale has been increasingly supported by clinical data across multiple tumor types, where combinations of antiangiogenic agents and ICIs have demonstrated meaningful antitumor activity. In thymic malignancies, early clinical studies have also suggested potential synergy between these therapeutic approaches. The CAVEATT phase 2 study showed that avelumab plus axitinib achieved an ORR of 34% in patients with unresectable or metastatic type B3 thymoma and thymic carcinoma, although immune‐related AEs remained an important consideration [18]. More recently, the PECATI phase 2 study demonstrated promising activity of lenvatinib plus pembrolizumab in pretreated advanced B3 thymoma and thymic carcinoma, with a median PFS of 14.9 months and manageable but clinically relevant immune‐related toxicity [19]. Together, these findings support further investigation of antiangiogenic‒ICI combinations in thymic malignancies. Given lucitanib's multitargeted inhibition of VEGFR, FGFR, and PDGFR pathways, future studies evaluating lucitanib in combination with ICIs may help define whether this strategy can further improve outcomes for patients with advanced thymic carcinoma.

Several limitations of this study should be acknowledged. The modest sample size, inherent to trials in rare malignancies, limited statistical power and may have contributed to baseline imbalances despite randomization. In addition, the absence of stratified randomization and prespecified stratified log‐rank analyses restricted adjustment for prognostic covariates and likely contributed to the violation of the proportional hazards assumption, thereby reducing the sensitivity of conventional log‐rank testing. Although multiple exploratory sensitivity analyses consistently favored lucitanib, these analyses were not prespecified and were not adjusted for multiplicity. Therefore, their results should be interpreted as supportive evidence that may help contextualize the primary analysis rather than as confirmatory findings. Besides, the proportional hazards assumption was not satisfied for IRC‐assessed PFS based on a post hoc Schoenfeld residual‐based assessment. Therefore, the interpretation of the estimated HR and log‐rank test requires caution, as these methods may not fully capture treatment effects that vary over time. Complementary analyses that are less dependent on the proportional hazards assumption provided supportive evidence of a potential treatment benefit with lucitanib. Finally, the Bayesian analysis was conducted post hoc as a supplementary sensitivity analysis and was not prespecified. Although non‐informative priors were employed to minimize subjective bias and ensure data‐driven posterior estimates, formal prior sensitivity analyses were not performed. Furthermore, our Bayesian survival modeling evaluated both Weibull and log‐normal models. Because the Weibull model presupposes a constant HR over time rather than adjusting for non‐proportional hazards, the resulting posterior probability of HR < 1 should be interpreted with caution as a model‐based inference.

In conclusion, lucitanib demonstrated clinically meaningful improvements in treatment response and disease control, with an acceptable safety profile, in patients with advanced recurrent or metastatic thymic carcinoma. As the first randomized, placebo‐controlled study in this setting, these findings support lucitanib as a promising therapeutic option in the second‐line or later treatment of this rare and aggressive malignancy.

## 4 Methods

### 4.1 Study Design and Participants

This was a prospective, randomized, double‐blind, placebo‐controlled phase 2 trial conducted at 14 study centers in China (Table S6). Eligible patients met the following inclusion criteria: age 18‒75 years; histologically or cytologically confirmed advanced recurrent or metastatic thymic carcinoma; failure of at least one line of chemotherapy; ineligibility for surgical resection or radical radiotherapy; at least one measurable lesion according to Response Evaluation Criteria in Solid Tumors (RECIST) version 1.1; an ECOG performance status score of 2 or less; adequate hematologic, hepatic, and renal function; body weight of at least 40 kg; and an estimated life expectancy of at least 12 weeks, as determined by the investigator.

Patients were excluded if they met any of the following criteria: unresolved AEs from prior anticancer therapy exceeding grade 1 according to the Common Terminology Criteria for Adverse Events (CTCAE), with the exception of alopecia and oxaliplatin‐related peripheral neuropathy of grade 2 or less; a history of another malignant tumor within the previous 5 years; the presence of central nervous system metastases requiring clinical intervention or tumor‐related epilepsy; receipt of chemotherapy, molecular targeted therapy, immunotherapy, biologic therapy, hormone therapy, traditional Chinese anticancer medicine, or other systemic anticancer treatment within 2 weeks before the first dose of study drug; receipt of radical radiotherapy (including radiotherapy involving more than 25% of the bone marrow) within 4 weeks before the first dose, or palliative radiotherapy for bone metastases within 2 weeks before the first dose; prior treatment with VEGFR TKIs or programmed death 1 (PD‐1) or programmed death ligand 1 (PD‐L1) inhibitors; clinically uncontrolled serous cavity effusions; bleeding events of CTCAE grade 3 or higher or bleeding tendencies within 4 weeks before treatment initiation; active duodenal ulcer, ulcerative colitis, intestinal obstruction, or other conditions associated with a risk of gastrointestinal bleeding or perforation; or a history of unhealed intestinal perforation or fistula.

The study was registered with ClinicalTrials.gov (NCT07824505) and the Chinese Clinical Trial Registry (CTR20180996). The protocol was approved by the independent ethics committee or institutional review board at each participating center and was conducted in accordance with the principles of the Declaration of Helsinki and Good Clinical Practice guidelines. All patients provided written informed consent before enrollment.

### 4.2 Procedures

Patients with advanced recurrent or metastatic thymic carcinoma who had failed at least one line of chemotherapy underwent centralized randomization via an interactive web response system, in a 2:1 ratio, to receive lucitanib at a dose of 10 mg once daily (lucitanib group) or placebo. Investigators, the sponsor, and patients remained blinded to treatment allocation. Best supportive care was provided in both groups. Each treatment cycle was defined as 4 weeks, and treatment was continued until disease progression, unacceptable toxicity, death, or discontinuation of study treatment for any other reason. For patients who were unable to tolerate the assigned treatment because of AEs, up to two dose reductions were permitted (7.5 mg once daily; 5 mg once daily).

Tumor assessments were performed by investigators and an IRC using computed tomography or magnetic resonance imaging in accordance with RECIST version 1.1 at baseline, every 4 weeks during the first two treatment cycles, and every 8 weeks thereafter until disease progression, withdrawal of consent, loss to follow‐up, death, or the end of study. Confirmation of response was performed using the same imaging method 4 weeks after the initial documentation of partial response (PR) or complete response (CR). After disease progression, patients were followed up for subsequent tumor therapy and survival every 12 weeks until death, study termination, or study completion. AEs were evaluated at screening, throughout treatment, and for up to 28 days after the final dose of study treatment, according to the CTCAE version 4.03.

### 4.3 Outcomes

The primary endpoint was PFS, as assessed by the IRC according to RECIST version 1.1. PFS was defined as the time from randomization to the first documented disease progression or death from any cause, whichever occurred first.

Secondary endpoints included investigator‐assessed PFS according to RECIST version 1.1, the 6‐month PFS rate, ORR, DCR, DoR, OS, and safety. ORR was defined as the proportion of patients with a best overall response of CR or PR. DCR was defined as the proportion of patients with a best overall response of CR, PR, or stable disease lasting at least 6 weeks. DoR was defined, among patients who achieved CR or PR, as the time from the first documented objective response to disease progression or death, whichever occurred first. OS was defined as the time from randomization to death from any cause. Safety assessments included AEs, clinical laboratory tests, electrocardiograms, and vital signs.

### 4.4 Statistical Analysis

The sample size was determined based on the primary endpoint of PFS using an event‐driven design. We assumed that lucitanib would improve the median PFS from 2 months in the placebo group [20, 21, 22] to 6 months in the lucitanib group [8, 23], corresponding to an HR of 0.33. Using a two‐sided significance level of 0.05, a statistical power of 90%, and a 2:1 randomization ratio, the required number of PFS events was calculated as 40 using the log‐rank test. Assuming an accrual period of 17 months and an additional follow‐up period of 6 months after enrollment of the last patient, approximately 48 patients were expected to yield the required number of PFS events. Considering an anticipated dropout rate of 12.5%, a total of 54 patients were planned for enrollment, including 36 patients in the lucitanib group and 18 patients in the placebo group. During study conduct, enrollment was extended beyond the initially estimated sample size because the observed accrual of IRC‐confirmed PFS events was slower than anticipated following the amendment of the primary endpoint assessment and the impact of the COVID‐19 pandemic.

Analyses of PFS, ORR, DCR, and OS were conducted in the full analysis set (FAS), defined as all randomized patients who received at least one dose of the study drug. In accordance with the intention‐to‐treat principle, patients in the FAS were analyzed according to their randomized treatment assignment. Safety analyses included all patients who received at least one dose of lucitanib or placebo.

PFS and OS were compared between treatment groups using the log‐rank test. The Kaplan‒Meier method was used to estimate the distributions of PFS, OS, and DoR, and to calculate median event times with corresponding two‐sided 95% CIs for each treatment group. Cox proportional hazards models were used to estimate HRs and 95% CIs for PFS and OS. Forest plots were used to display subgroup analyses of PFS comparing the lucitanib and placebo groups. For ORR and DCR, 95% CIs were calculated using the Clopper‒Pearson method. The maximum percentage change from baseline in the sum of target lesion diameters for each patient was illustrated using waterfall plots.

In addition, because the proportional hazards assumption was violated for IRC‐assessed PFS when evaluated using conventional methods, alternative statistical approaches that are less sensitive to this assumption were applied in post hoc sensitivity analyses. These included weighted log‐rank tests using the Gehan‒Breslow, Tarone‒Ware, Peto‒Peto, and modified Peto‒Peto methods, which assign greater weight to early progression events [24, 25, 26, 27]. RMST analysis was also performed to estimate average PFS over prespecified time horizons and to mitigate bias arising from between‐group risk differences at the tail of the Kaplan‒Meier curves in the setting of a small sample size. In addition, Bayesian regression analyses assuming log‐normal and Weibull distributions were conducted, with goodness‐of‐fit assessments, and the posterior probability of lucitanib being superior to placebo was estimated.

## Author Contributions

**Wentao Fang and Caicun Zhou**: conceptualization and methodology. **Wentao Fang, Changlu Wang, Kailiang Wu, Jianhua Fu, Xiaoyan Lin, Helong Zhang, Jianhua Chen, Peng Zhang, Yiping Zhang, Peng Tang, Yulong Zheng, Yanping Liu, Xiuyi Zhi, Zhancheng Gao, and Qingxia Fan**: data curation and formal analysis. **Huiming Cai**: writing – original draft. **Fugen Li and Xiaoning Guo**: supervision. **Bo Zhang**: project administration. **Rongji Mu and Zhangsheng Yu**: formal analysis. All the authors have read and approved the final manuscript.

## Funding

This study was sponsored by Haihe Biopharma Co., Ltd. The funder provided support in the form of salaries for authors Huiming Cai, Fugen Li, Xiaoning Guo, and Bo Zhang, and was involved in supervision of the study conduct, project administration, guidance on data analysis and interpretation, and manuscript preparation through these authors. The decision to submit the manuscript for publication was made by all authors. The specific roles of these authors are articulated in the “Author contributions” section.

## Ethics Statement

The study protocol was approved by the Ethics Committee of Shanghai Chest Hospital (approval no. LS1817). The trial was prospectively registered in the Chinese Drug Trial Registry (registration no. CTR20180996) and retrospectively registered at ClinicalTrials.gov (NCT07824505). The delay in ClinicalTrials.gov registration was administrative in nature and was unrelated to study design, regulatory approval, or trial implementation. The study was conducted in accordance with the principles of the Declaration of Helsinki and Good Clinical Practice guidelines. Written informed consent was obtained from all patients before enrollment.

## Conflicts of Interest

Huiming Cai, Fugen Li, Bo Zhang, and Xiaoning Guo are employed by Haihe Biopharma Co., Ltd. The rest of authors have no conflicts of interest to declare.

## Supporting information



Supporting File 1: mco271105‐sup‐0001‐SuppMat.docx

## Data Availability

The authors confirm that the data supporting the findings of this study are available within the article and its Supporting Information. Other study data may be shared upon submission of a request to corresponding author.
