# Supplementary material for: Efficacy and Safety of Lucitanib in Advanced Thymic Carcinoma: A Randomized Phase II Trial
Source: MedComm (2020). 2026 Oct 25;7(11):e71105. doi: 10.1002/mco2.71105 (PMC13695807; doi:10.1002/mco2.71105)
Supplement: Supplementary file 1 [file MCO2-7-e71105-s001.docx]

**Supplementary materials for**

Efficacy and Safety of Lucitanib in Advanced Thymic Carcinoma: A Randomized Phase II Trial

Wentao Fang^1*†^, Caicun Zhou^2†^, Changlu Wang^3^, Kailiang Wu^4^, Jianhua Fu^5^, Xiaoyan Lin^6^, Helong Zhang^7^, Jianhua Chen^8^, Peng Zhang^9^, Yiping Zhang^10^, Peng Tang^11^, Yulong Zheng^12^, Yanping Liu^13^, Xiuyi Zhi^14^, Zhancheng Gao^15^, Qingxia Fan^16^, Huiming Cai^17^, Fugen Li^17^, Xiaoning Guo^17^, Bo Zhang^17^, Rongji Mu^18^, Zhangsheng Yu^18^

1. Department of Thoracic Surgery, Shanghai East Hospital, Shanghai Tong Ji University School of Medicine, Shanghai, China

2. Department of Medical Oncology, Shanghai East Hospital, Shanghai Tong Ji University School of Medicine, Shanghai, China

3. Department of Radiation Oncology, Shanghai Chest Hospital, Shanghai Jiao Tong University School of Medicine, Shanghai, China

4. Radiotherapy Center, Fudan University Shanghai Cancer Center, Shanghai, China

5. Department of Thoracic Surgery, Sun Yat-sen University Cancer Center, Guangzhou, China

6. Department of Medical Oncology, Fujian Medicine University Union Hospital, Fuzhou, China

7. Department of Oncology, The Second Affiliated Hospital of Air Force Medical University, Xi’an, China

8. Department of Thoracic Medicine, Hunan Cancer Hospital, Changsha, China

9. Department of Cardiothoracic Surgery, Tianjin Medicine University General Hospital, Tianjin, China

10. Department of Medical Oncology, Zhejiang Cancer Hospital, Hangzhou, China

11. Department of Esophageal Oncology, Tianjin Cancer Hospital, Tianjin, China

12. Department of Medical Oncology, The First Affiliated Hospital, Zhejiang University School of Medicine, Hangzhou, China

13. Department of Oncology, China Shenyang The Tenth People's Hospital, Shenyang, China

14. Department of Thoracic Surgery, Xuanwu Hospital Capital Medicine University, Beijing, China

15. Department of Respiratory Medicine, Peking University People's Hospital, Beijing, China

16. Department of Medical Oncology, The First Affiliated Hospital of Zhengzhou University, Zhengzhou, China

17. Haihe Biopharma Co., Ltd, Shanghai, China

18. Institute of Clinical Medicine, Shanghai Jiao Tong University School of Medicine, Shanghai, China

^†^Contributed equally to the study as co-first authors.

*Correspondence to: Prof Wentao Fang, Department of Thoracic Surgery, Shanghai East Hospital, Shanghai Tong Ji University School of Medicine, 150 Jimo Road, Pudong District, Shanghai, China, E-mail: [vwtfang@hotmail.com](mailto:vwtfang@hotmail.com)

**The file includes:**

Supplementary Methods

Figure S1 to S2

Table S1 to S6

# Figure S1. Bayesian Analyses for Progression-Free Survival Assessed by IRC.

Bayesian Regression with Goodness-of-fit tests from log-normal distribution and Weibull distribution were used for IRC-assessed PFS, and the corresponding posterior probability of lucitanib being superior to placebo was reported. Bayesian analyses showed that the posterior probability of the lucitanib group over the placebo group in IRC-assessed PFS was approximately 100% (Pr (β1＞0∣D)=1), based on Bayesian Log-Normal Regression (Figure 4A), and the posterior probability was approximately 96.10% (Pr (HR＜1∣D)=0.961), based on Bayesian Weibull Regression (Figure 4B). Pr, probability; HR, hazard ratio; D, distribution.

**Figure S2. Kaplan-Meier Analysis of Progression-Free Survival and Overall Survival According to Dose Modification Status in the Lucitanib Group.**

(A) Progression-free survival assessed by the independent review committee (IRC). The median progression-free survival was 7.23 months in the dose-modification group and 2.87 months in the non-modification group (hazard ratio [HR], 0.35; 95% CI, 0.18-0.67). (B) Progression-free survival assessed by investigators. The median progression-free survival was 7.49 months in the dose-modification group and 4.67 months in the non-modification group (hazard ratio [HR], 0.35; 95% CI, 0.17-0.70). (C) Overall survival. The median overall survival was 17.68 months in the dose-modification group and 9.53 months in the non-modification group (hazard ratio [HR], 0.57; 95% CI, 0.27-1.17). Dose modification was defined as dose reduction or interruption.

**Table S1. Subsequent treatment and outcomes**

|  | Lucitanib (N=46) | Placebo (N=22) |
| --- | --- | --- |
| Subsequent anti-tumor therapy, n (%) | 27 (58.7) | 18 (81.8) |
| Regimen type, n (%) |  |  |
| Chemotherapy | 12 (26.1) | 10 (45.5) |
| Immunotherapy | 15 (32.6) | 8 (36.4) |
| Targeted therapy | 5 (10.9) | 3 (13.6) |
| Biotherapy |  |  |
| Others | 12 (26.1) | 3 (13.6) |
| Median treatment duration (days) (Q1, Q3) | 88.0 (41.0, 340.0) | 220.5 (35.0, 509.0) |
| Best overall response, n (%) |  |  |
| CR | 0 | 0 |
| PR | 1 (2.2) | 0 |
| SD | 2 (4.3) | 0 |
| PD | 3 (6.5) | 2 (9.1) |
| Unknown | 18 (39.1) | 14 (63.6) |
| Not applicable | 3 (6.5) | 2 (9.1) |
| Reasons for treatment discontinuation, n (%) |  |  |
| Disease progression | 4 (8.7) | 2 (9.1) |
| Drug toxicity | 0 | 0 |
| Completion of treatment regime | 6 (13.0) | 6 (27.3) |
| Patient’s decision | 4 (8.7) | 2 (9.1) |
| Others | 7 (15.2) | 3 (13.6) |

**Table S2. Reasons for deaths**

|  | Lucitanib (N=46) | Placebo (N=22) |
| --- | --- | --- |
| Death, n (%) | 31 (67.4) | 10 (45.5) |
| Death more than 28 days after the last dose | 30 (65.2) | 10 (45.5) |
| Death within 28 days of the last dose | 1 (2.2) | 0 |
| Primary cause of death, n (%) |  |  |
| Disease progression | 22 (71.0) | 4 (40.0) |
| Adverse event | 1 (3.2) | 0 |
| Unknown | 8 (25.8) | 6 (60.0) |

**Table S3. Multivariable Cox Proportional Hazards Regression Analyses of Progression-Free Survival**

| Analysis | Endpoint | Covariate | Hazard ratio (95% CI) | P value |
| --- | --- | --- | --- | --- |
| Prespecified analysis | IRC-assessed PFS | Lucitanib vs placebo | 0.58 (0.30-1.11) | 0.1008 |
|  |  | Histological type (squamous cell carcinoma vs others) | 0.51 (0.22-1.17) | 0.1110 |
|  |  | Surgical resection (performed vs not performed) | 1.12 (0.61-2.04) | 0.7108 |
|  |  | Radiotherapy (administered vs not administered) | 0.87 (0.45-1.71) | 0.6950 |
| Prespecified analysis | Investigator-assessed PFS | Lucitanib vs placebo | 0.49 (0.26-0.90) | 0.0220 |
|  |  | Histological type (squamous cell carcinoma vs others) | 0.59 (0.26-1.35) | 0.2144 |
|  |  | Surgical resection (performed vs not performed) | 1.22 (0.67-2.24) | 0.5124 |
|  |  | Radiotherapy (administered vs not administered) | 0.84 (0.43-1.65) | 0.6169 |
| Exploratory post hoc analysis | IRC-assessed PFS | Lucitanib vs placebo | 0.45 (0.24-0.86) | 0.0161 |
|  |  | Time from initial diagnosis to randomization | 0.98 (0.96-1.00) | 0.0140 |
| Exploratory post hoc analysis | Investigator-assessed PFS | Lucitanib vs placebo | 0.44 (0.24-0.80) | 0.0078 |
|  |  | Time from initial diagnosis to randomization | 0.99 (0.97-1.00) | 0.0636 |

IRC, independent radiologic review committee; PFS, progression-free survival.

# Table S4. Response to Treatment as Evaluated by Investigator and IRC (Full Analysis Set)

|  | **Lucitanib Group**  **(*n* = 46)** | **Placebo Group (*n* = 22)** |
| --- | --- | --- |
| Assessed by IRC |  |  |
| Best overall response *n* (%) |  |  |
| Partial response | 7 (15.2) | 0 |
| Stable disease | 31 (67.4) | 11 (50.0) |
| Progressive disease | 4 (8.7) | 9 (40.9) |
| Not evaluable^*^ | 3 (6.5) | 2 (9.1) |
| Objective response rate (95% CI) | 15.2 (6.3, 28.9) | 0 (0.0, 15.4) |
| Disease control rate (95% CI) | 82.6 (68.6, 92.2) | 50.0 (28.2, 71.8) |
| Duration of response, months, median (95%CI) | 7.1 (3.48, 10.71) | - |
| Assessed by investigator |  |  |
| Best overall response *n* (%) |  |  |
| Partial response | 6 (13.0) | 0 |
| Stable disease | 33 (71.7) | 10 (45.5) |
| Progressive disease | 5 (10.9) | 11 (50.0) |
| Not evaluable^*^ | 1 (2.2) | 1 (4.5) |
| Objective response rate (95% CI) | 13.0 (4.9, 26.3) | 0 (0.0, 15.4) |
| Disease control rate (95% CI) | 84.8 (71.1, 93.7) | 45.5 (24.4, 67.8) |
| Duration of response, months, median (95%CI) | 6.5 (3.68, NE） | - |

^*^ Patients receiving study treatment for a duration of less than 6 weeks.

IRC, independent review committee; CI, confidence interval; NE, not estimated

**Table S5. Overall study drug exposure (SAS)**

|  | Lucitanib (N=46) | Placebo (N=22) |
| --- | --- | --- |
| Cumulative weeks of treatment, weeks, median (range) | 24.20 (1.9, 110.7) | 8.20 (3.6, 48.7) |
| Cumulative weeks of treatment categories |  |  |
| < 8 weeks | 9 (19.6) | 8 (36.4) |
| 8-24 weeks | 12 (26.1) | 8 (36.4) |
| > 24 weeks | 25 (54.3) | 6 (27.3) |
| Cumulative number of treatment cycles, median (range) | 7 (1, 28) | 3 (1, 13) |
| Cumulative dose (mg), median (range) | 1593.75 (130.0, 7300.0) | 575.00 (250.0, 3400.0) |
| Actual dose intensity (mg/day), mean (SD) | 8.75 (1.515) | 9.87 (0.424) |
| Relative dose intensity (%), mean (SD) | 87.46 (15.146) | 98.62 (4.250) |
| Relative dose intensity categories |  |  |
| < 80% | 14 (30.4) | 0 |
| 80%-120% | 32 (69.6) | 22 (100.0) |
| > 120% | 0 | 0 |
| Patients with at least one dose modification | 29 (63.0) | 6 (27.3) |
| Dose reduction | 21 (45.7) | 1 (4.5) |
| AE | 21 (45.7) | 1 (4.5) |
| Others | 1 (2.2) | 0 |
| Dose interruption | 29 (63.0) | 6 (27.3) |
| AE | 26 (56.5) | 3 (13.6) |
| Others | 15 (32.6) | 6 (27.3) |
| Time from first dose to first dose reduction (days), median (range) | 102.0 (15, 372) | 131.0 (131, 131) |
| Time from first dose to first dose interruption (days), median (range) | 57.0 (3, 360) | 47.5 (8, 310) |
| Cumulative duration of dose interruption (days), median (range) | 18.0 (1, 62) | 2.5 (1, 20) |

**Table S6. Ethical approval numbers of the participating centers**

| **Ethics Committee** | **Approval number** |
| --- | --- |
| Shanghai Chest Hospital Ethics Committee | LS1817 |
| Fudan University Shanghai Cancer Center Medical Ethics Committee | 1808189-4-1812 |
| Sun Yat-sen University Cancer Center Ethics Committee | A2018-068-01 |
| Fujian Medicine University Union Hospital Ethics Committee | 2018YW015-02 |
| Tangdu Hospital, Fourth Military Medical University Medical Ethics Committee | No. 201806-15 |
| Hunan Cancer Hospital Medical Ethics Committee | 2018 Fast Review No. (158) |
| Tianjin Medicine University General Hospital | IRB2018-044-01 |
| Zhejiang Cancer Hospital Medical Ethics Committee | IRB-(2019)301 |
| Tianjin Cancer Hospital Medical Ethics Committee | E2018112 |
| The First Affiliated Hospital, Zhejiang University School of Medicine, Medical Ethics Committee | 2018 EC No. (128) |
| China Shenyang The Tenth People's Hospital Medical Ethics Committee | 2018-005-01 |
| Xuanwu Hospital Capital Medicine University Ethics Committee | Clinical Drugs review (2018) No. 014 |
| Peking University People's Hospital Medical Ethics Committee | 2018PHA075-001 |
| The First Affiliated Hospital of Zhengzhou University Research and Clinical Trial Ethics Committee | Drugs-2018-40 |
